# Regression of *BRAF*^V600E^ mutant adult glioblastoma after primary combined BRAF-MEK inhibitor targeted therapy: a report of two cases

**Published:** 2019-06-04

**Authors:** Peter Y.M. Woo, Tai-Chung Lam, Jenny K.S. Pu, Lai-Fung Li, Roland C.Y. Leung, Jason M.K. Ho, James T.F. Zhung, Belinda Wong, Timonthy S.K. Chan, Herbert H.F. Loong, Ho-Keung Ng

**Affiliations:** ^1^ Department of Neurosurgery, Kwong Wah Hospital, Hong Kong; ^2^ Department of Clinical Oncology, Li Ka Shing Faculty of Medicine, The University of Hong Kong, Hong Kong; ^3^ Department of Neurosurgery, Queen Mary Hospital, Hong Kong; ^4^ Department of Medicine, Queen Mary Hospital, Hong Kong; ^5^ Department of Neurosurgery, Kwong Wah Hospital, Hong Kong; ^6^ Pharmacy and Medical Therapeutics, Kwong Wah Hospital, Hong Kong; ^7^ Department of Pathology, Kwong Wah Hospital, Hong Kong; ^8^ Department of Clinical Oncology, The Chinese University of Hong Kong, Hong Kong; ^9^ Department of Anatomical and Cellular Pathology, The Chinese University of Hong Kong, Hong Kong

**Keywords:** glioblastoma, BRAF^V600E^ mutation, targeted therapies, BRAF-MEK inhibitors

## Abstract

**Background:**

Up to 15% of young adults with glioblastoma have the activating oncogenic *BRAF*^V600E^ mutation, an actionable target of the MAPK signal transduction pathway governing tumor cell proliferation. Small molecule inhibitors of BRAF and MEK, a downstream protein immediately following BRAF, have been shown to confer a survival advantage for patients with *BRAF*^V600E^ mutant advanced melanoma. We describe our experience using this combined target therapy for two patients with BRAF^V600E^ mutant glioblastoma (GBM) as primary treatment due to extenuating clinical circumstances that prohibited the prescription of standard treatment.

**Case Presentation:**

The two patients were both 22 years old on presentation. After the initial tumor resection, they both developed rapid deterioration in performance status within a few weeks due to leptomeningeal metastases. In view of the critical condition, BRAF and MEK inhibitors were prescribed as first line treatment. The two patients both achieved dramatic clinical response, which was parallel to the impressive radiological regression of the disease. Unfortunately, the duration of disease control was short as drug resistance developed rapidly. The two patients died 7 and 7.5 month after initial diagnosis of GBM.

**Conclusions:**

Primary treatment with inhibitors of BRAF and MEK can lead to tumor regression for patients with *BRAF*^V600E^ mutant glioblastoma. We therefore recommend that all young GBM patients should undergo BRAF^V600E^ mutation testing, especially for those with unusual aggressive clinical course.

## INTRODUCTION

Glioblastoma is one of the most aggressive of adult brain malignancies and carries a poor prognosis. In spite of standard treatment, comprising of surgical resection followed by concomitant temozolomide chemo-radiotherapy (CCRT), overall survival is limited to 14.6 months [1]. The discovery of epigenetic methylguanine-methyltransferase (MGMT) promoter methylation as a prognostic-predictive biomarker has significantly guided clinical decision-making [2]. However, no further breakthrough in management has been made for more than a decade and is partially contributed by the failure to translate promising laboratory findings into clinical practice.

The RAS-RAF-MEK-ERK (MAPK) signaling pathway is principally responsible for the cellular response to various membrane-based receptor tyrosine kinases (RTK). The pathway is chiefly activated by various growth signals mediated by epidermal growth factor receptors (EGFR) or platelet-derived growth factor receptors (PDGFR) that have been identified to be integral in malignant gliomagenesis and cell proliferation [3]. Oncogenic driver mutations of the MAPK pathway’s signaling components, for example the RAF protein family, of which BRAF has the highest intrinsic kinase activity, has frequently been detected in various human cancers such as melanoma [4]. In particular, the missense constitutively active V600E type mutation of the *BRAF* oncogene (*BRAF^V600E^*) is commonly associated with pediatric gliomas [5]. The discovery that small molecule inhibitors targeting BRAF (BRAFi) and its downstream MEK protein (MEKi) improved overall survival (OS) among *BRAF^V600E^* mutant melanoma patients has precipitated interest in its application in primary central nervous system tumors [6, 7]. *BRAF^V600E^* mutation was found to be relative common among pediatric gliomas compared with adults with favorable responses having been reported for both high and low gliomas [6].

We report our experience in treating two young adults that had *BRAF^V600E^* mutant glioblastoma with combined BRAFi/MEKi inhibitor therapy as first-line treatment. In both patients disease progressed rapidly soon after presentation which precluded the use of conventional therapy. Both patients had considerable tumor regression after initiating treatment illustrating the potential clinical relevance of target therapy in the management of this subset of glioblastomas.

## CASE PRESENTATION

### Patient 1

**FIGURE 1:**
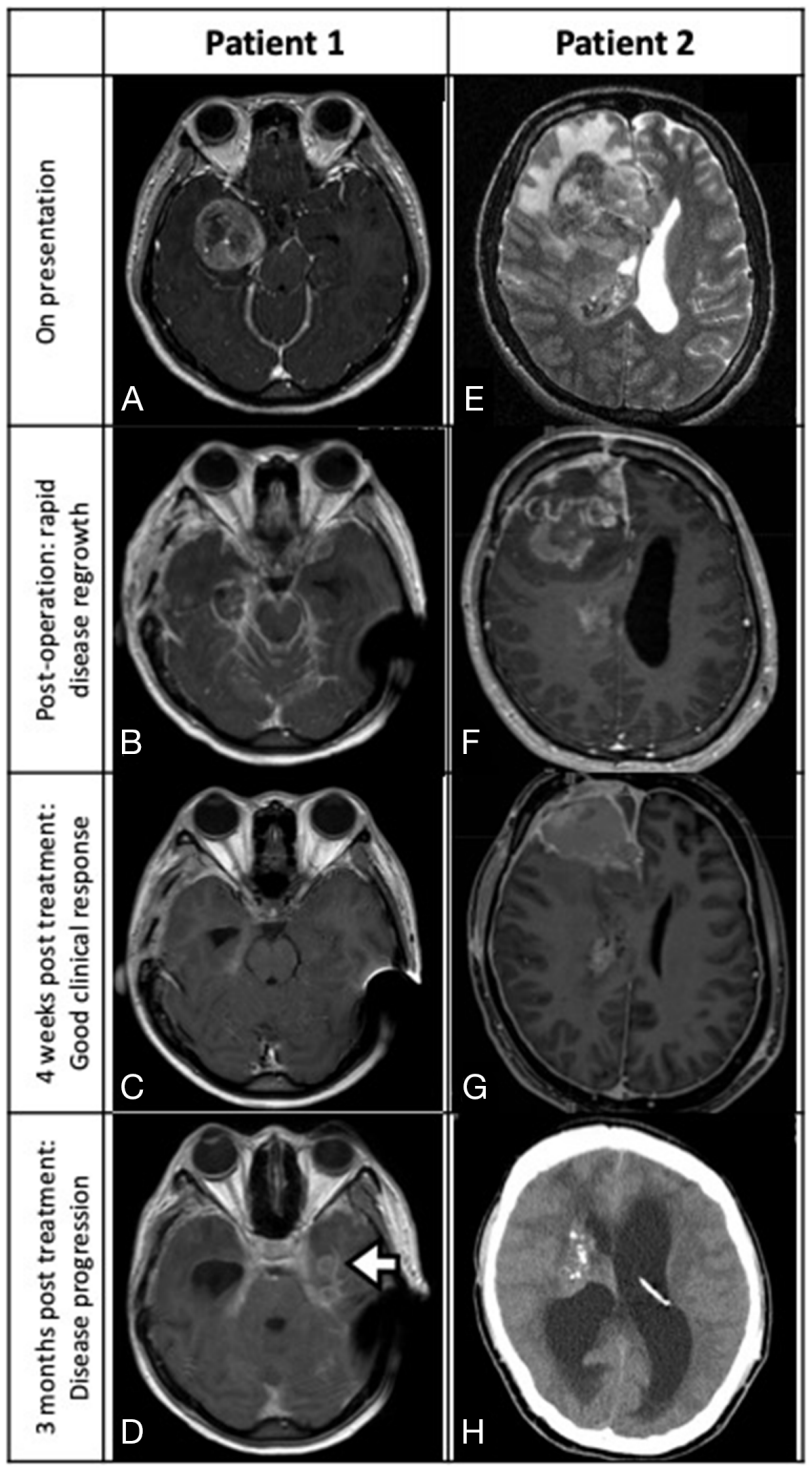
**Patient 1 (A-D)**: MRI depicting a right temporal glioblastoma with ambient cistern LMS (A, axial T1-weighted contrast-enhanced sequence). Post-near total resection three-week MRI showing local recurrence with diffuse LMS (B, axial). After receiving four weeks of dabrafenib and trametinib, significant tumor regression was noted (C, axial MRI). Three months after starting combined target therapy, LMS with a new left temporal lesion was detected (D, axial MRI; white arrow, multifocal tumor recurrence). **Patient 2 (E-H):** MRI scan revealing a right frontal glioblastoma with spread into the body of the right lateral ventricle (E, axial T2-weighted sequence; white arrowhead, ventricular tumor). Post-subtotal resection MRI showing rapid regrowth of tumor at surgical cavity and the development of communicating hydrocephalus (F, sagittal T1-weighted contrast-enhanced sequence). Significant tumor regression observed four weeks after starting vemurafenib (G, axial T1 contrast enhanced MRI). Disease rapidly progressed after stopping BRAF inhibitor and developed severe hydrocephalus (H, plain CT).

A 22 year-old woman presented with headache for three months with a preoperative Karnofsky performance score (KPS) of 90. Magnetic resonance imaging (MRI) revealed a heterogeneous contrast enhancing right temporal intra-axial tumor (3.2cm x 3.6cm x 4.0cm) with evidence of leptomeningeal spread (LMS) at the right ambient cistern (Figure 1A). Craniotomy with near total excision was performed and the histological diagnosis was an isocitrate dehydrogenase-1 (IDH-1) wildtype, MGMT promoter methylated epithelioid glioblastoma with *BRAF^V600E^* mutation. During the early postoperative period the patient rapidly developed communicating hydrocephalus that required ventriculoperitoneal (VP) shunting. Within a week the shunt became blocked with tumor-fibrin clots that required external ventricular drainage (EVD). A three-week MRI scan revealed focal tumor recurrence with diffuse intracranial and cervical spinal cord LMS (Figure 1B). The patient’s consciousness deteriorated to a Glasgow Coma Score (GCS) of 10/15 (E2V3M5) and her KPS dropped to 30 requiring nasogastric tube feeding. Given the patient’s poor neurological state and her reliance on EVD, temozolomide CCRT was not considered possible. Because of the *BRAF^V600E^* mutation findings, combined dabrafenib 150mg BD and trametinib 4mg daily systemic therapy was started. A single session of whole brain radiotherapy (3Gy) was also administered with the aim to enhance blood brain barrier drug permeability. The patient had considerable clinical improvement two weeks after treatment initiation with full recovery of consciousness. She was able to wean off the EVD and nasogastric tube. Three weeks after starting combined BRAFi/MEKi therapy a MRI revealed substantial tumor regression (Figure 1C). The patient largely tolerated the target therapy experiencing grade II cutaneous adverse reactions. After a course of rehabilitation the patient was discharged home with a KPS of 80. Tumor tissue targeted gene panel analysis was performed by next-generation sequencing (NGS) and the results are summarized in Table 1.

**Table 1:** Summary table of NGS targeted gene panel for patients 1 and 2

	Patient 1	Patient 2
**Primary Tumor NGS Panel**		
Tumor purity	55%	58%
BRAF V600E	Allele frequency 56.6%	Allele frequency 40.8%
Retinoblastoma protein	Wild type	Wild type
CDKN2A, CDKN2B	Homozygous deletion	Homozygous deletion
PTEN	Heterozygous deletion	No deletion
CHEK1	Heterozygous deletion	No deletion
BRCA1	Heterozygous deletion	No deletion
NF1	Heterozygous deletion	No deletion
Microsatellite Instability	MSI-low	MSS
Total mutation load (mutations per megabase)	17.1	6.0
**CSF Cell-free DNA NGS Panel Upon Radiological Recurrence**		
BRAF V600E	72.0%	35.7%

After three months of combined target therapy the patient developed progressive neck pain within a week. She also suffered from rapid weight loss and deterioration in KPS to 50. An MRI revealed a recurrent lesion in the contralateral mesial temporal lobe with LMS over the cervical and upper thoracic cord (Figure 1D). CSF collected for cytology showed malignant tumor cells and next generation sequencing of CSF for cell-free DNA found high levels of *BRAF^V600E^* mutant DNA indicating acquired treatment resistance. Since the tumor cells harbored a borderline high mutational load (17.1 mutations per megabase) with low microsatellite instability (only one of the five mononucleotide repeat markers of the pentaplex polymerase chain reaction panel was positive), the PD-1 checkpoint inhibitor nivolumab was added concurrently with the BRAFi/MEKi therapy (Table 1). Whole brain radiotherapy was not given due to the rapid deterioration in functional performance and for concerns that the simultaneous administration of BRAFi therapy could cause severe neurotoxicity and cutaneous adverse reactions. The patient’s condition continued to deteriorate and palliative spinal radiotherapy (15Gy over five fractions) was finally prescribed. In spite of such salvage treatments there was further disease progression and the patient died seven months after diagnosis.

### Patient 2

A 22 year-old man with good past health and G6PD deficiency presented with a two-month history of headache. A MRI brain (Figure 1E) showed a 5.4 x 5.8 x 5.2cm right frontal lobe contrast-enhancing intra-axial tumor that extended into the right lateral ventricle. Craniotomy with subtotal resection was performed with CSF specimens revealing the presence of tumor cells. The pathological diagnosis was an epithelioid glioblastoma (IDH-1 wildtype, MGMT promoter methylated) with as high as 20 mitotic figures detected per ten high power field. Further molecular tests showed TERT mutation and absence of EGFR amplification. NGS targeted gene panel testing confirmed the presence of *BRAF^V600E^* mutation (Table 1).

Originally temozolomide CCRT was planned, but the patient rapidly developed a focal tumor recurrence, diffuse LMS and communicating hydrocephalus that required VP shunting (Figure 1F). With a KPS of only 40 the patient was considered physically unfit for chemo-irradiation and was prescribed the BRAFi, vemurafenib 960mg BD (dabrafenib was not used due to G6PD deficiency). After only two days of treatment, the patient reported a significant alleviation of his headache and a three-week MRI confirmed significant tumor regression (Figure 1G). The MEKi, cobimetinib (60mg daily) was subsequently added. The patient’s clinical condition improved considerably reaching a KPS of 80 and he was discharged home after a short course of rehabilitation. He tolerated the combined target therapy and only developed a grade II photosensitivity rash. A four-week MRI scan showed good treatment response with partial tumor regression (Figure 1G).

In anticipation that tumor resistance could arise as with our first patient, standard temozolomide CCRT was started. Vemurafenib and cobimetinib were stopped one week beforehand to minimize the risk of cutaneous photosensitivity and neurotoxicity. But only after one week of CCRT the patient developed severe neck pain with a computed tomography (CT) scan revealing local recurrence and hydrocephalus (Figure 1H). Chemo-irradiation was stopped with revision of the VP shunt performed. Combined target therapy was resumed 10 days later, but the patient continued to have multiple episodes of shunt occlusion due to the elevated CSF tumor-fibrin clot load and eventually required an EVD. CSF cell-free DNA testing also detected persistently high levels of *BRAF^V600E^* mutated DNA that signified possible drug resistance (Table 1). The patient was not considered fit for resumption of CCRT therefore a selective cyclin-dependent kinase 4/6 inhibitor, palbociclib was started at 75mg daily, 60% of maximum dose, for 21 days per 28 days cycle, concurrently with vemurafenib 960mg BD. Two weeks later an endoscopic third ventriculostomy was performed and the patient was able to wean off the drain.

The patient tolerated this new target therapy combination well and resulted in a pronounced recovery of consciousness although his overall KPS remained poor at 40. Whole brain radiotherapy was not resumed due to the patient’s poor performance status and concerns that synergistic adverse effects could arise when given concomitantly with BRAFi therapy. Follow-up CT scanning showing stable disease and palbociclib was stepped up to 100 mg for the second cycle. However, the treatment response was transient and after eight weeks of therapy the patient developed severe intratumoral hemorrhage and succumbed resulting in an overall survival of 7.5 months.

## DISCUSSION

BRAF-MEK dual node inhibition of the MAPK pathway in our two young adult glioblastoma patients achieved significant, albeit transient, clinical and radiological response. To our knowledge this is the first report in the literature describing the use of combined BRAFi/MEKi therapy for *BRAF^V600E^* mutant glioblastomas as primary treatment (Table 2).

**Table 2:** Reported cases in the literature of *BRAF*^V600E^ mutant glioblastoma patients treated with BRAFi target therapy

Author / Year	Age (years) / Sex	Tumor Location	Histology / Molecular Profile	Treatment	Time from Target Therapy Initiation to Treatment Response Detection by MRI (Weeks)	OS (months)
Robinson et al / 2014^21^	9 / M	Fronto-parietal lobe	Epithelioid - *BRAF^V600E^* mutation	*Primary:* Radiotherapy with vorinostat (radiosensitizer) + Bevacizumab *Recurrence:* Vemurafenib 720mg BD	8	> 36
Arvantis et al / 2014	40 / F	Temporal	Epithelioid - *BRAF^V600E^* mutation	*Primary:* TMZ CCRT and adjuvant TMZ *1^st^ recurrence:* SRS + Bevacizumab *2^nd^ recurrence:* Vemurafenib	8	N.A.
Leaver et al / 2016	26 / M	Temporal lobe with LMS and pulmonary metastases	*BRAF^V600E^* mutation	*Primary:* Vemurafenib 960mg BD	1	< 2
Burger et al / 2017	25 /M	Temporal lobe	Non-epithelioid - IDH-1 wildtype MGMT promoter methylation inconclusive *BRAF^V600E^* mutation	*Primary:* TMZ CCRT and adjuvant TMZ *1^st^ recurrence with LMS:* Lomustine *2^nd^ recurrence:* Dabrafenib 150mg BD	1	> 3
Abadal et al / 2017	34 / F	Parietal lobe	Non-epithelioid - IDH-1 wildtype MGMT promoter methylation No EGFR amplification *BRAF^V600E^* mutation	*Primary:* SRS + TMZ CCRT and adjuvant TMZ Intolerance to TMZ: switched to bevacizumab *1^st^ recurrence with LMS:* Vemurafenib	4	> 22
Ceccon et al / 2018	19 / M	Temporo-parietal lobe	Epithelioid Secondary (previous anaplastic astrocytoma) *-* IDH-1 wildtype *BRAF^V600E^* mutation	*Primary:* Interstitial brachytherapy^125^ I-seeds + Radiotherapy *1^st^ recurrence:* TMZ CCRT and adjuvant TMZ *4^th^ recurrence:* Lomustine *5^th^ recurrence:* Dabrafenib 150mg BD	N.A.	103
Current study / 2018	22 / F 23 / M	Temporal lobe LMS Frontal lobe with LMS	Epithelioid - IDH-1 wildtype MGMT promoter methylation No EGFR amplification *BRAF^V600E^* mutation Epithelioid - IDH-1 wildtype MGMT promoter methylation No EGFR amplification *BRAF^V600E^* mutation Homozygous deletion of CDKN2A and CDKN2B	*Primary:* Dabrafenib 150mg BD + Trametinib 4mg daily *1^st^ recurrence*: Nivolumab 100mg daily *Primary:* Vemurafenib 960mg BD + Cobimetinib 60mg daily Switched to TMZ CCRT *1^st^ recurrence*: Vemurafenib 960mg BD + Pablociclib 100mg daily	3 3	7 7.5

N.B. BRAFi, BRAF inhibitor; OS, overall survival; LMS, leptomeningeal spread; IDH-1, isocitrate dehydrogenase-1; TMZ, temozolomide; CCRT, concomitant chemoradiotherapy; MGMT, methylguanine methyltransferase; SRS, stereotactic radiosurgery; EGFR; epidermal growth factor receptor; N.A., not available.

Due to extenuating clinical circumstances standard temozolomide CCRT was not prescribed for our two patients. Off-label use of BRAF and MEK inhibitors was attempted as a treatment of last resort. The initial response was dramatic with significant tumor regression observed as early as three weeks with minimal toxicity. This remarkable response is echoed by similar observations in the treatment of *BRAF^V600E^* mutated melanoma [8].

Our strategy to forestall the possibility of acquired tumor resistance to target therapy for Patient 2 by pre-emptively switching to standard temozolomide CCRT failed to achieve a clinical response. Disease progression was particularly aggressive and Patient 2 required several CSF diversion surgeries within two weeks of target therapy withdrawal. His swift deterioration exemplifies the potentially high chemo- and radio-resistance of recurrent *BRAF^V600E^-*mutated glioblastoma. However, as with melanomas where most patients relapse within months, our observations also suggest that BRAFi/MEKi resistance precludes long-term survival.

In previous reports, *BRAF^V600E^* mutations are rarely encountered in adult glioblastoma with an incidence ranging from less than 1% to 8% [5, 9, 10]. But young adults, i.e. 17 to 35 years, seem to have higher mutation rates of up to 15% [11]. Controversy exists on whether a *BRAF^V600E^* mutation constitutes a favorable biomarker for glioblastoma. A review of young adult glioblastoma patients revealed that those with *BRAF* mutant tumors had a median OS of 43.2 months compared to their wild-type counterparts with a median OS of 13.6 months [12]. This is supported by the high occurrences of this mutation among lower grade gliomas: 66% in pleomorphic xanthoastrocytomas, 18% in gangliogliomas and 9% in pilocytic astrocytomas [5]. However, 50% of epithelioid glioblastomas, a more aggressive variant compared to classic glioblastoma, also harbor *BRAF^V600E^* mutations with patients having a significantly shorter median OS of only 5.6 months [13, 14]. Epithelioid glioblastomas typically affect younger patients and generally expressing early recurrence with leptomeningeal spread, features that closely resembled the clinical course of our patients [15]. This in contrast to previous reports that have observed that for non-epithelioid glioblastomas, *BRAF^V600E^* mutated glioblastomas might confer a better prognosis [6, 12].

The FDA approved vemurafenib, a BRAFi, in 2011 for the treatment of *BRAF^V600E^*-mutant melanomas after the results of a phase III randomized-controlled trial revealed improved OS [7]. But the durability of such treatment was short-lived since acquired resistance to monotherapy was observed as early as six months after treatment initiation with paradoxical reactivation of the MAPK pathway as the predominant mechanism [16]. Since MEK is an immediate downstream protein from RAF, trametinib was developed as a selective inhibitor in combination with BRAFi. Randomized trials confirmed that by adopting this strategy of dual-node pathway suppression with combined BRAFi/MEKi treatment, a 25% risk reduction in disease progression was observed compared to BRAFi therapy alone [17, 18]. This culminated in the FDA approval in 2014 of combined BRAFi/MEKi therapy, dabrafenib and trametinib, for *BRAF^V600E^* mutant unresectable or metastatic melanomas. In particular, survival benefit was noted among patients with melanoma brain metastasis, which are notoriously resistant to radiotherapy, and illustrated the ability of these small molecule inhibitors to cross the BBB [11]. With these encouraging results a few reports have described the therapeutic potential of off-label MAPK pathway target therapy for recurrent *BRAF^V600E^* mutant pediatric gliomas [19-24].

Our experience demonstrates that without standard radiotherapy, small molecule inhibitors not only can traverse the BBB, but also could result in considerable glioblastoma regression. With BRAFi/MEKi therapy we encountered an earlier than expected remarkable treatment response that was similar with other reports (Table 2). Five glioblastoma patients, all of them 40 years old or younger, have been described in the literature where BRAFi monotherapy was given as second line salvage treatment and only one as primary treatment [21, 22, 25-28].

Although tumor regression was seen in our patients, it was transient and progression-free survival was only three months. The resistance to combined BRAFi/MEKi therapy is a big unmet clinical need and the mechanism of resistance is under heavy investigations. Currently three main categories of mutations were identified as the cause of MEK/ERK signaling pathway. The first category is BRAF gene amplification[29]. In melanoma study, it affects 36% of patients treated with combined BRAFi/MEKi therapy. This leads to increased BRAF kinase concentration in the cell, creates an excess of activated MEK and hence elevates MAPK signaling[30]. The second category is the de novo mutations at MEK1/2 which may account ~25% of treatment resistance[31, 32]. Most of these mutations occur within, or close to the ATP binding site which may allosterically increases the intrinsic kinase activity of MEK[32]. Thirdly, de novo mutation of NRAS may involve a gain-of-function point mutation, most commonly at codon 61, 12 or 13[33]. This leads to a subsequent hyperactivation of the RAS-RAF-MAPK and P13KT-AKT cascades[34]. Beyond genomic aberrations, transcriptional alternations including elevated Nuclear Factor-κB (NF-κB) transcriptional activity[35], c-MET up expression, infra-physiologic LEF1 down expression and YAPI signature enrichment[33, 36] were all possible mechanisms of resistance to combined therapy. In the current report, high level of BRAF mutant DNA was detected in the CSF of our two patients when they developed clinical evidence of disease progression, while other mutations (NRAS, MEK) were not detected. So BRAF amplification was a possible cause of resistance in these two patients.

## CONCLUSIONS

Combined target therapy as first-line treatment could potentially benefit selected *BRAF^V600E^* mutant glioblastoma patients who have rapid disease progression and are unfit for temozolomide CCRT. We recommend that young glioblastoma patients, i.e. younger than 30 years, should undergo *BRAF^V600^* mutation testing either by direct sequencing or immunohistochemistry [37]. Further study is required to address the acquisition of resistance, but this report indicates that combined target therapy may have a significant role in the limited armamentarium against glioblastoma.
